# Homozygous CRISPR/Cas9 Knockout Generated a Novel Functionally Active Exon 1 Skipping XPA Variant in Melanoma Cells

**DOI:** 10.3390/ijms231911649

**Published:** 2022-10-01

**Authors:** Veronika Banicka, Marie Christine Martens, Rüdiger Panzer, David Schrama, Steffen Emmert, Lars Boeckmann, Alexander Thiem

**Affiliations:** 1 Clinic and Policlinic for Dermatology and Venereology, University Medical Center Rostock, 18057 Rostock, Germany; 2 Department of Dermatology, Venereology and Allergology, University Hospital Würzburg, 97080 Würzburg, Germany

**Keywords:** DNA repair, nucleotide excision repair, XPA, CRISPR, knockout, protein variant, melanoma, A375

## Abstract

Defects in DNA repair pathways have been associated with an improved response to immune checkpoint inhibition (ICI). In particular, patients with the nucleotide excision repair (NER) defect disease Xeroderma pigmentosum (XP) responded impressively well to ICI treatment. Recently, in melanoma patients, pretherapeutic XP gene expression was predictive for anti-programmed cell death-1 (PD-1) ICI response. The underlying mechanisms of this finding are still to be revealed. Therefore, we used CRISPR/Cas9 to disrupt XPA in A375 melanoma cells. The resulting subclonal cell lines were investigated by Sanger sequencing. Based on their genetic sequence, candidates from *XPA* exon 1 and 2 were selected and further analyzed by immunoblotting, immunofluorescence, HCR and MTT assays. In *XPA* exon 1, we established a homozygous (*c.19delG*; p.A7Lfs*8) and a compound heterozygous (*c.19delG/c.19_20insG*; p.A7Lfs*8/p.A7Gfs*55) cell line. In *XPA* exon 2, we generated a compound heterozygous mutated cell line (*c.206_208delTTG/c.208_209delGA*; p.I69_D70delinsN/p.D70Hfs*31). The better performance of the homozygous than the heterozygous mutated exon 1 cells in DNA damage repair (HCR) and post-UV-C cell survival (MTT), was associated with the expression of a novel XPA protein variant. The results of our study serve as the fundamental basis for the investigation of the immunological consequences of XPA disruption in melanoma.

## 1. Introduction

In the last years, the defects of different DNA repair pathways have been associated with an increased response to immune checkpoint inhibition (ICI) in cancer patients [1,2,3]. Certainly, the most prominent clinical example is the first site/tissue agnostic approval of pembrolizumab by the U.S. food and drug administration for mismatch repair (MMR) deficient (or microsatellite instability-high) cancer [4]. Besides MMR, there are two other pathways that repair DNA single-strand breaks (SSB): base excision repair (BER); and nucleotide excision repair (NER). Remarkably, the immunological consequences of NER defects are far less explored than those of MMR and BER, although people suffering from the NER defect disease, xeroderma pigmentosum (XP), had an impressive response of their skin tumors to anti-programmed cell death-1 (PD-1) ICI in case reports [2,5,6,7,8,9,10,11,12]. Of note, we just recently revealed that expression of XP genes was predictive of response in two cohorts of anti-PD-1 treated melanoma patients [13]. However, the fundamental basis of our observation is not yet clear and needs further investigation by, e.g., cell experiments. We therefore used Clustered Regularly Interspaced Short Palindromic Repeats (CRISPR) and CRISPR-associated (Cas) protein 9 to target XPA, the central coordinator of NER and scaffold provider for other NER proteins, in the well-established human melanoma cell line A375 [14,15,16,17].

CRISPR/Cas9 is an innovate DNA-editing system, which was originally discovered as a prokaryotic adaptive immunity mechanism for the cleavage of invading nucleic acids [18,19,20,21]. When utilizing in genome engineering, specific single guide (sg)RNAs are designed to bind to a DNA target sequence in the gene of interest. Complementary pairing of a sgRNA to its target results in the generation of a blunt-end double strand break (DSB) by Cas9 [18,21]. In eukaryotic cells, those are predominately repaired by error-prone non-homologous end joining (NHEJ) and to a lesser extent by more precise homology driven repair (HDR) [18,21]. NHEJ simply re-ligates the two strands without a homologous template and thereby often generates insertions and/or deletions (InDels) at the cleavage site [22]. Hence, it usually fails in restoring the original genetic sequence, leading to nonsense sequences, rendering the resulting protein inactive or at least less active. NHEJ is also fundamental for CRISPR/Cas9-mediated gene knockouts [21,23,24]. Upon targeting one site in a coding exon, knockouts arise from InDel-mediated frameshift mutations. They characteristically introduce premature stop codons, which disrupt gene expression or can lead to proteins missing the N-terminal region due to alternative translation initiation (ATI) [21,24].

By applying CRISPR/Cas9, we generated three different *XPA*-mutated A375 subclonal cell lines. Targeting exon 1 resulted in two knockout cell lines for wild type XPA—a homozygous (*c.19delG;* p.A7Lfs*8) and a compound heterozygous (*c.19delG/c.19_20insG*; p.A7Lfs*8/p.A7Gfs*55)—both leading to short N-terminal protein fragments. By targeting exon 2, we established a compound heterozygous cell line bearing an in-frame 3-base pair deletion in one of the two *XPA* alleles, while having a frameshifting mutation on the other (*c.206_208delTTG/c.208_209delGA*; p.I69_D70delinsN/p.D70Hfs*31). Notably, homozygous XPA exon 1 knockout resulted in expression of a novel XPA protein variant, which was associated with an improved survival following UVC irradiation compared to the other two cell lines.

## 2. Results

### 2.1. CRISPR/Cas9 Application

**Figure 1:**
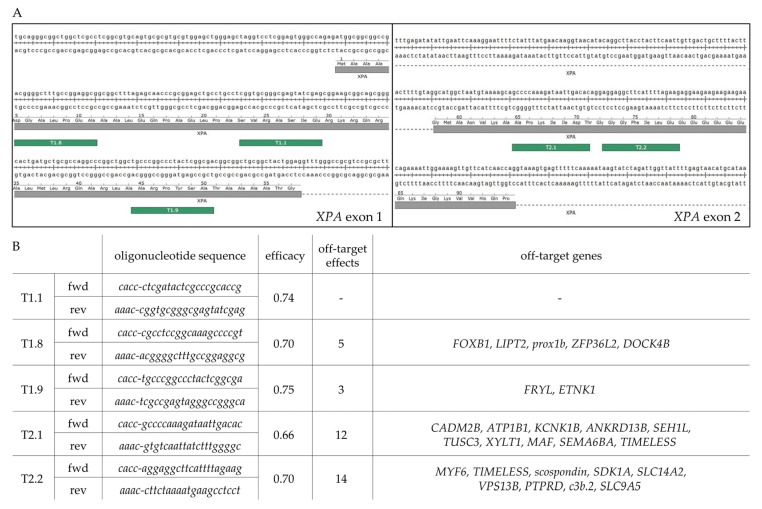
Selection process of sgRNAs. (**A**) Overview of chosen XPA exon 1 and 2 target sequences (represented by green bars) as suggested by CRISPR/Cas9 target online predictor (CCTop, University of Heidelberg) and their location within the exonic sequences modified from SnapGene Viewer, GSL Biotech LLC, San Diego, CA. Coding region represented by grey bars. (**B**) Characterization of the five final sgRNAs. Target sequences with prespecified off-target effects directly related to DNA repair and cell survival were excluded. The selection was based on high efficacy and/or low number of off-target effects. sgRNA nomenclature included their targeted exon followed by a consecutive number. Oligonucleotide fwd- and rev- sequences are shown including their overhangs, CACC and AAAC, for cloning into the BbsI cleavage site in PX459. Fwd, forward (5′–3′); Rev, reverse (3′–5′); sgRNA, single-guide RNA.

**Figure 2:**
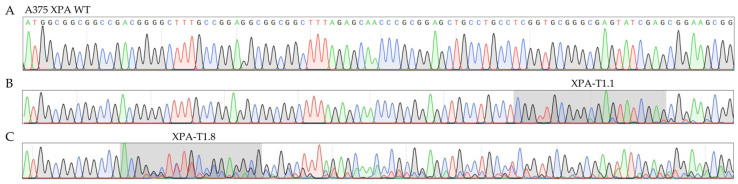
Sanger sequencing chromatograms comparing *XPA* WT (**A**) and *XPA* polyclonal cells targeted with sgRNAs T1.1 (**B**) or T1.8 (**C**) in exon 1. The latter were chosen for the generation of single cell clones, whereas T1.1 targeted cells were discarded. The gray regions highlight the DNA sequences targeted by the respective sgRNAs. Four-color chromatogram depicting the four DNA bases adenine (green), cytosine (blue), guanine (gray), and thymine (red). sgRNA, single-guide RNA; WT, wildtype.

For the CRISPR/Cas9-mediated knockout of XPA, we used PX459 plasmids encoding five sgRNAs (T1.1, T1.8, T1.9, T2.1 and T2.2), targeting the first two exons of the XPA gene. After transfection into A375 melanoma cells and puromycin selection [25] (Figure 1), PCR sequencing of the targeted exons confirmed the generation of polyclonal XPA-mutated cell lines (Figure 2).

In this context, those polyclonal cell lines were considered particularly promising, that included the most additional superimposed peaks in the sequencing chromatogram (Figure 2, auspicious XPA-T1.8 compared to barely altered XPA-T1.1). Two polyclonal cell lines (targeted with T1.8 in exon 1 and with T2.1 in exon 2) were finally chosen to generate stable subclones bearing mutations on both XPA alleles through single clone expansion.

### 2.2. Establishment of Stable XPA-Mutated Cell Lines

**Figure 3:**
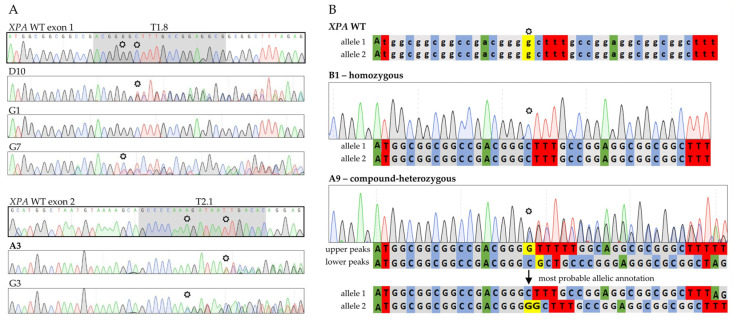
Analysis of CRISPR/Cas9-generated single cell-derived lines. (**A**) Sanger sequencing chromatograms of *XPA* exon 1 (targeted with sgRNA T1.8) and exon 2 (targeted with sgRNA T2.1) illustrating the different outcomes. Monoclonal G1 cells remained unaltered, while all other depicted subclones carried mutations on both alleles. WT DNA regions targeted with the two different sgRNAs are highlighted in gray (**B**) Sequencing chromatograms of *XPA* WT and *XPA* exon 1 knockouts with depiction of underlying alleles. *Stars* indicate mutation start sites in the subclones as well as the original bases in the WT. Selected cell lines written in bold. The detailed description of the allelic assignment process can be found in the Material and Methods Section 4.3. *sgRNA*, single-guide RNA; *WT*, wildtype; *A*, adenine (green); *C*, cytosine (blue); *G*, guanine (gray); *T*, thymine (red).

Subclonal single cell lines, named after their well of origin, were analyzed by Sanger sequencing of the targeted XPA exons (Figure 3A,B). Manifest genetic outcomes included subclones containing the wildtype (G1), or mutations on one or both alleles in the targeted region. Biallellic mutations were either homozygous (exon 1: B1) or compound-heterozygous (exon 1: A9, D10 and G7; exon 2: A3 and G3).

Based on the Sanger sequencing results, two exon 1 knockout cell lines bearing frameshifting mutations on both alleles leading to early stop codons—the homozygous B1 (c.19delG) and the compound-heterozygous A9 (c.19delG/c.19_20insG)—were selected for further investigations (Figure 3B). From the exon 2 targeted subclones we chose the compound heterozygous A3 subclone (c.206_208delTTG/c.208_209delGA).

### 2.3. XPA Expression

**Figure 4:**
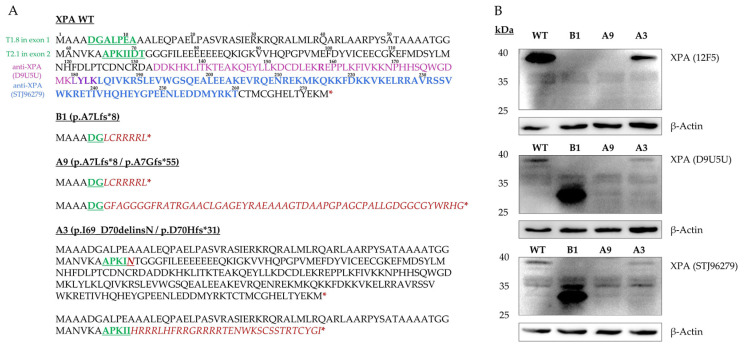
Protein analysis of the three XPA mutated subclonal cell lines compared to XPA WT. (**A**) Predicted AA sequences of homozygous (WT, B1) and heterozygous (A9, A3) cell lines following the regular transcription and translation start sites are depicted. The XPA WT is based on NCBI transcript CCDS6729. AA sequences corresponding to the sgRNA-targeted regions are shown in green, altered AA sequences in red. Stars indicate translation termination sites upon the first in-frame stop codon. The approximate epitopes of the anti-XPA antibodies are highlighted for D9U5U (purple, targeting ≈ 50 AA surrounding the published target AA R158) and STJ96279 (blue) clones. The epitope of the 12F5 antibody is not published. (**B**) Immunoblotting with the 12F5 antibody showed complete loss of full length XPA in exon 1 knockout cell lines, B1 (p.A7Lfs*8) and A9 (p.A7Lfs*8/p.A7Gfs*55), while still producing a weaker XPA signal in A3 (p.I69_D70delinsN/p.D70Hfs*31) cells. Using D9U5U and STJ96279, a smaller band was newly detected in B1, presumably representing a de novo XPA variant. The AA one-letter-code was used in both panels. AA, amino acid(s); sgRNA, single-guide RNA; WT, wildtype.

The genetic code of both alleles was used to predict the order of amino acids (AA) of the most likely resulting proteins presuming that transcription and translation start sites were unaltered (Figure 4A). DNA alterations in exon 1 targeted cell lines, B1 (c.19delG) and A9 (c.19delG/c.19_20insG), caused frameshift mutations leading to premature stop codons (p.A7Lfs*8; p.A7Lfs*8/p.A7Gfs*55) and thereby short N-terminal protein fragments. Only the first six AA were predicted to correspond to the XPA wildtype. Accordingly, in both exon 1 knockout subclones, immunoblotting revealed a loss of the ~37 kDa protein band when incubated with the monoclonal 12F5 XPA antibody (unpublished epitope; Figure 4B, upper panel). Our exon 2-mutated cells (A3) were anticipated to bear an in-frame mutation on one allele and a frameshift mutation on the other (p.I69_D70delinsN/p.D70Hfs*31). Consistently, the 12F5 antibody recognized XPA, though the signal was much weaker (Figure 4B, upper panel). Of note, when the homozygous exon 1 knockout cells (p.A7Lfs*8) were incubated with the monoclonal D9U5U antibody (targets residues surrounding R158) or the polyclonal STJ96279 antibody (binds C-terminally at AA 180–260), a new band of approximately ~31–33 kDa was detected (Figure 4B, middle and bottom panel).

We hypothesized, that this constituted a de novo XPA protein variant missing the N-terminal start of the wildtype protein, and explored its functional consequences following UVC irradiation.

### 2.4. UV-Induced DNA Damage and Its Repair

**Figure 5:**
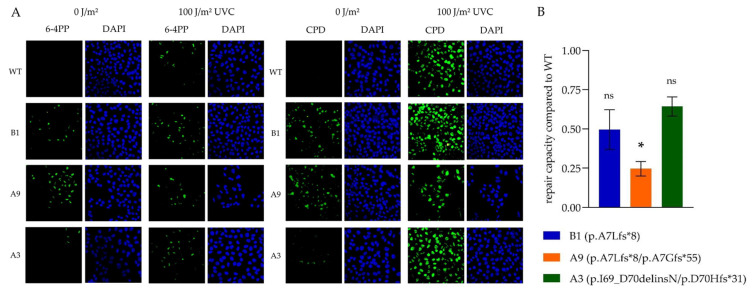
UVC-damage accumulation and repair. (**A**) Representative immunofluorescence of non-irradiated cells or cells 90 mins after irradiation with 100 J/m^2^ UVC stained with anti-CPD and anti-6-4PP antibodies. DAPI was used as a nuclear control. The XPA WT cell line showed DNA damage only after irradiation, while it was detected even at baseline in the CRISPR/Cas9-altered subclones. All pictures captured with a 40x objective lens. (**B**) In the HCR assay, repair of irradiated firefly luciferase plasmids in relation to the baseline luminescence activity of non-irradiated firefly plasmids is depicted for the three subclonal cell lines compared to XPA WT (set as value 1). All subclonal cell lines demonstrated impaired DNA damage repair capacity, which was statistically significant only for A9. The means ± SEM of three independent experiments are depicted. A significance is indicated by star (* *p* < 0.05; one-way-ANOVA). *6-4PPs*, 6-4 pyrimidine pyrimidone photoproducts; *AA*, amino acids; *CPDs*, cyclobutane pyrimidine dimers; *DAPI*, 4′,6-diamidino-2-phenylindole; *HCR*, host-cell-reactivation; *ns*, not significant; *SEM*, standard error of mean; *WT*, wildtype.

6-4 pyrimidine pyrimidone photoproducts (6-4PPs) and cyclobutane pyrimidine dimers (CPDs) are the main DNA lesions repaired by NER and they were analyzed by immunofluorescence before and after UV-C irradiation [16] (Figure 5A). XPA wildtype (WT) showed DNA defects only after irradiation with UVC, while 6-4PPs and CPDs were surprisingly detected even in non-irradiated B1 and A9 knockout cells. In exon 2-mutated A3, only a weak basal DNA damage signal could be detected. 100 J/m^2^ UVC irradiation produced clearly visible DNA damage in all tested cell lines, however, it was most detrimental to A9, characterized by lower cell numbers observed after irradiation.

In the host-cell reactivation (HCR) assay, the repair of the irradiated (1000 J/m^2^ UVC) firefly plasmid was measured and set in relation to the baseline luminescence activity of non-irradiated firefly plasmids. All subclones had a diminished repair compared to the WT (Figure 5B). In general, repair of both exon 1 mutated cell lines was reduced to a greater extent. Among each other, repair in homozygous B1 cells was less impaired than in its heterozygous counterpart A9, possibly due to the expression of a compensatory new XPA protein variant.

### 2.5. Post-UVC Metabolic Activity Indicating Cell Survival

**Figure 6:**
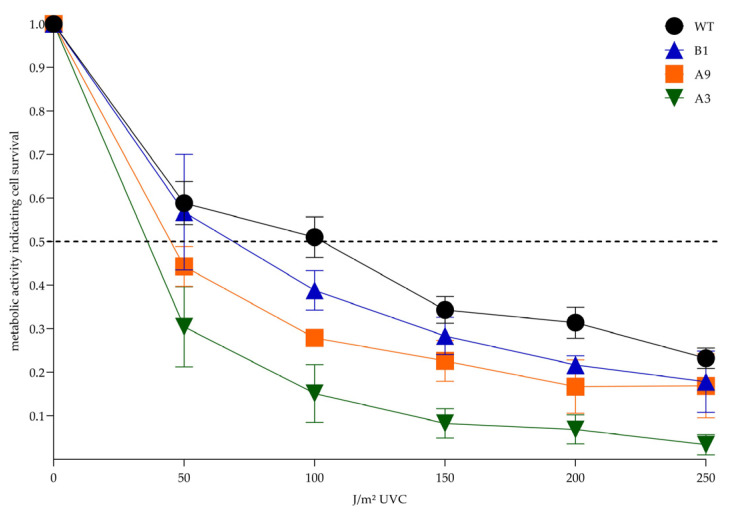
Post-UVC metabolic activity indicating cell survival. In the MTT assay all CRISPR/Cas9-generated subclones were more sensitive to the different UVC doses than the parental A375 XPA WT cells, reflected by a steeper gradient of the descending metabolic activity curves. Statistically significant differences between WT and the generated subclones were observed for A3 cells at doses of 100 J/m^2^ and 200 J/m^2^ (*p* = 0.03 and *p* = 0.04, respectively; one-way-ANOVA). A *p*-value < 0.05 was regarded as statistically significant. The dotted line represents the LD_50_. The mean values ± SEM of 3 independent experiments are depicted. LD_50_, lethal dose, 50%; MTT, 3-(4,5-dimethylthiazol-2-yl)-2,5-diphenyltetrazolium bromide tetrazolium; WT, wildtype.

Next, we systematically assessed the metabolic activity of subclonal cell lines compared to the XPA wildtype in the (3-(4,5-dimethylthiazol-2-yl)-2,5-diphenyltetrazolium bromide) tetrazolium (MTT) assay after irradiation with increasing doses of UVC (0–250 J/m^2^), that have been previously determined by our dosis-finding experiments. Again, the CRISPR/Cas9-generated subclones were more sensitive to UVC than the parental A375 XPA WT cells. Since the MTT assay is commonly used for determination of cell survival, we calculated that 50% of the WT cells were still living after 101 J/m^2^ UVC (defined as their lethal dose [LD]_50_), while LD_50_ doses were much lower in the CRISPR/Cas9-altered cell lines (Figure 6). Importantly, from the exon 1 knockout cell lines, homozygous B1 cells were again less affected by UVC irradiation than heterozygous A9 cells. Remarkably, metabolic activity was most restricted in A3 cells with a significant reduction compared to the WT at doses of 100 J/m^2^ and 200 J/m^2^.

## 3. Discussion

Using CRISPR/Cas9, we generated several single cell-derived A375 subclonal cell lines, in which we successfully disrupted both XPA alleles within its first two exons. From the cells targeted in exon 1, we chose two similarly mutated knockout cell lines. While B1 cells were homozygously altered lacking one guanine (G) on either XPA allele (c.19delG), A9 cells had one allele identically mutated as B1 and an inserted G on the other (c.19delG/c.19_20insG) (Figure 3B). These alterations, provided translation start at the regular site, caused frameshift mutations resulting in premature termination codons (PTCs) and thereby leading to short N-terminal XPA protein fragments. However, functional consequences following UVC irradiation differed between B1 and A9. In this regard, better performance of B1 cells in HCR (Figure 5B) and MTT (Figure 6) assays was consistent with the expression of a novel smaller XPA protein variant, that we detected in our immunoblot analysis (Figure 4B).

CRISPR/Cas may have diverse on-target impacts on protein level rather than exclusive knockout [23,24]. After introduction of frameshift mutations, the fate of subsequent mRNAs differs. First, mRNAs can be recognized by the cell as aberrant and degraded by nonsense mediated decay (NMD) [24,26,27]. Second, if a transcript bearing frameshifting mutations resists NMD, it can be translated in a truncated protein. Third, during RNA processing, alternative splicing (AS) events may occur, including particularly skipping of exons affected by mutations [24]. These splice variants can still provide partial functionality of the original protein or produce proteins with alternate functions. Fourth, ATI beginning at other start codon can give rise to various new protein variants [23,26,27].

For our B1 cells, all these possible mechanisms leading to different protein variants were taken into consideration. mRNA expression was assessed by qPCR with primers targeting exon 1 and exons 2–3 (Supplementary Figure S1A). NMD was deemed implausible since WT and B1 had overlying amplification plots of exon 1 specific primers (Supplementary Figure S1B). With the other primer pair, we analyzed a second section of the mRNA transcript sequence downstream of the mutation-bearing exon 1 and compared its expression with expression of the first target. Provided similar PCR efficiency, WT cells revealed a similar expression of both primer targets, while B1 cells expressed significantly more of the second target (Supplementary Figure S1C,D). This is consistent with the expression of a second transcript lacking exon 1, possibly due to AS. In addition, B1 DNA sequence was scanned for possible downstream ATI sites, revealing in-frame start codons in exon 1 (M37) and exon 2 (M59) (Figure 4A). Notably, according to predictions with the *Peptide Mass Calculator* (available at www.peptidesynthetics.co.uk/tools, accessed on 30 September 2022) a novel B1 protein variant, possibly originating from AS or ATI or even both, and lacking the mutated region of exon 1, would match with the approx. −4 to −6 kDa smaller protein detected by XPA antibody clones D9U5U and STJ96279 (Figure 4B).

A3 cells (p.I69_D70delinsN/p.D70Hfs*31) possessed a frameshift mutation only in one allele, whereas the other was predicted to encode for a slightly altered XPA-variant with two AA lacking and a new one inserted (Figure 4A). Accordingly, immunoblot with all three tested XPA antibodies detected a protein band, however, it was much weaker than in the WT (Figure 4B). Correspondingly, the load of basal DNA damage was the lowest in immunofluorescence (Figure 5A), and the DNA repair capacity was least reduced in the HCR assay (Figure 5B). Surprisingly, this cell line was massively impaired in the MTT assay (Figure 6), which is commonly used to investigate cellular survival.

The MTT assay reflects the cellular metabolic activity, which usually correlates with cell viability [28,29]. Nevertheless, several conflicting cases are documented in the literature showing that MTT is not always accurate in mapping cell numbers. Moreover, in some cases metabolism-independent cell viability assays contradicted those results generated by MTT [28,30,31,32]. Additionally, in XP patients clinical sun sensitivity and post-UV survival of patient’s cell lines, as assessed by MTT, did not always match with each other [33]. That is why we questioned whether the XPA mutations in A3 might influence metabolic activity and thereby could interfere with the MTT assay. The targeted mutation in A3, particularly the deletion of D70, occurred in a region of XPA exon 2, indispensable for recruitment and function of the endonuclease ERCC1 [14,15,17,34,35,36,37,38]. Indeed, ERCC1 depletion in cancer cells resulted in lower nicotinamide adenine dinucleotide phosphate hydrogen (NADPH), NADP+, NADH and NAD+ levels [39]. Because MTT assay is NAD(P)H-dependent, lower ERCC1 recruitment in A3 might explain their poor MTT performance [28,31].

The immunofluorescence was used to visualize 6-4PPs and CPDs as the main DNA lesions repaired by NER. Of note, in our CRISPR/Cas 9 altered cells a weak signal indicating DNA damage could be detected even without direct UVC irradiation. We assume that this DNA damage was acquired during the standardized handling of all cells including washing steps and mock irradiation. Importantly, this incidental UV exposure was not sufficient to cause detectable DNA lesions in wildtype cells, further supporting increased sensitivity of XPA mutated cells.

In our study, a transfection based CRISPR/Cas 9 approach was performed due to its lower probability of off-target effects [40]. Nevertheless, and as one limitation of our study, off-target effects may not be fully excluded. To this end, it would be necessary to sequence the whole genome of the generated subclonal cell lines. However, the most probable predicted exonic off-targets—to the best of our knowledge—do not influence the cellular functions analyzed. Furthermore, only one parental melanoma cell line (A375) was originally utilized in our study, though it was applied to generate multiple subclonal cell lines.

In conclusion, our study provides the first generation and detailed characterization of different CRISPR/Cas 9 XPA knockouts in A375 cells. In the context of our ICI-focused research, we will use these cell lines to investigate the immunological consequences of differently disrupted XPA proteins, including a novel XPA protein variant. Those experiments will involve the analysis of the expression of molecules, e.g., programmed-death-ligand 1 (PD-L1), that influences effective antitumor immune response and are thereby indispensable for the activity of anti-PD-1 ICI [41,42].

## 4. Materials and Methods

### 4.1. Cell Culture

A375 melanoma cells (CRL-1619) were obtained from the American Type Culture Collection (ATTC, Manassas, VA, USA) and cultured in Dulbecco’s Modified Eagle Medium (DMEM) (Thermo Fisher Scientific, Waltham, MA, USA) with 10% Fetal Bovine Serum (PAN Biotech, Aidenbach, Germany), 100 U/mL Penicillin and 100 μg/mL Streptomycin (Merck, Darmstadt, Germany) at 37  °C with 5% CO_2_. The six XPA exons of parental A375 were sequenced (Sanger Sequencing at Eurofins Genomic, Ebersberg, Germany; primer sequences are presented in Supplementary Table S1) to validate the wildtype. All cells were regularly tested for mycoplasma contamination.

### 4.2. Generation of sgRNA-PX459 Plasmids

For the design of single-guide (sg)RNAs targeting the first two exons of XPA, CRISPR/Cas9 target online predictor (CCTop, University of Heidelberg, available at https://cctop.cos.uni-heidelberg.de:8043/index.html, accessed on 30 September 2022) was used. Oligonucleotides were ordered from Sigma-Aldrich (Merck, Darmstadt, Germany). pSpCas9(BB)-2A-Puro (PX459) V2.0 was a gift from Feng Zhang (Addgene plasmid #62988; http://n2t.net/addgene:62988, accessed on 30 September 2022; RRID:Addgene_62988). PX459 was digested with BbsI and phosphorylated using Calf Intestinal Alkaline Phosphatase. Multiple single-stranded sgRNA oligonucleotide pairs were annealed at 95 °C, dephosphorylated using T4 Polynucleotide kinase and ligated into the PX459 plasmid (all enzymes from New England Biolabs, Ipswich, MA, USA). Ligation products were transformed by heat shock into chemocompetent DH5α Escherichia coli bacteria (New England Biolabs, Ipswich, MA, USA). Successful integration of the oligonucleotides in PX459 was verified by Sanger sequencing.

### 4.3. Generation of XPA-Mutated A375 Subclonal Cell Lines

The different sgRNA-PX459 plasmids were transfected into A375 cells (ViaFect Transfection Reagent, Promega, Madison, WI, USA), followed by selection with puromycin (InvivoGen, San Diego, CA, USA). Single-cell expansion of polyclonal lines was achieved by seeding of serially diluted cells in 96 well-plates. After DNA extraction (DNeasy, Qiagen, Hilden, Germany), Sanger sequencing was performed. For sequence analyses and sgRNA annotation SnapGene Viewer (GSL Biotech LLC, San Diego, CA, USA) was used. In case of heterozygous subclonal cell lines, superimposed peaks in the chromatogram were annotated as upper and lower peaks. For manual assignment, we analyzed those peaks searching for the wildtype sequence, that would follow an induced mutation after a possible insertion or deletion. Once identified, the peaks at each location were assigned to the alleles. The allele determinations were verified using the online tools DECODR, CRISPR-ID and Synthego (available at https://decodr.org/, http://crispid.gbiomed.kuleuven.be and https://ice.synthego.com, respectively. All accessed on 30 September 2022).

### 4.4. Immunofluorescence

A total of 75,000 cells/well were seeded on glass cover slips in 24 well-plates and incubated for 48 h. Cells were either irradiated with 100 J/m^2^ UVC (UVC 500 Crosslinker, Amersham Biosciences, Buckinghamshire, UK) or left non-irradiated. Prior to all irradiation experiments cells were washed with phosphate-buffered saline (PBS, PAN Biotech, Aidenbach, Germany) and all liquid was carefully removed before irradiation with UVC. DMEM was replaced and the cells were incubated 90 min before fixation with 4% PFA (Merck, Darmstadt, Germany) in PBS. They were probed with antibodies against 6-4PP (clone 64M-2, Abcam, Cambridge, UK) and CPD (clone TDM-2, Absolute Antibody, Cleveland, UK). The secondary anti-mouse antibody was AlexaFluor488-coupled (Thermo Fisher Scientific, Waltham, MA, USA). Cells were mounted with Fluoroshield Mounting Medium With DAPI (Abcam, Cambridge, UK) and analyzed with ZEISS Axio Imager.M2 (Carl Zeiss, Wetzlar, Germany).

### 4.5. Immunoblotting

Total cellular proteins were extracted at 4  °C using radioimmunoprecipitation assay (RIPA) buffer (Thermo Fisher Scientific, Waltham, MA, USA) containing protease inhibitors (Roche, Basel, Switzerland). Protein concentrations were determined using Pierce BCA assay kit (Thermo Fisher Scientific, Waltham, MA, USA) according to the manufacturer’s protocol. Proteins (10–20 μg) were resolved on 10% SDS–polyacrylamide gels and transferred to Amersham Protra 0.45  μm NC nitrocellulose membranes (Thermo Fisher Scientific, Waltham, MA, USA). They were probed with antibodies against XPA with three different antibody clones: 12F5 (Santa Cruz Biotechnology, Dallas, TX, USA), D9U5U (Cell Signaling Technology, Danvers, MA, USA) and STJ96279 (St John’s Laboratory, London, UK). β-Actin (detected by antibody clone AC-15, Merck, Darmstadt, Germany) served as loading control. Detection using horseradish peroxidase (HRP)-conjugated secondary antibodies (Agilent Dako, Santa Clara, CA, USA) was performed in the ECL Chemocam Imager (Intas Science Imaging Instruments, Göttingen, Germany).

### 4.6. Host Cell Reactivation (HCR) Assay

A total of 30,000 cells/well were seeded in 24 well-plates. After overnight incubation, XPA wildtype and CRISPR-rendered cells were transfected with non-irradiated renilla plasmid 50 ng/well (as transfection control), and either with non-irradiated or UVC-irradiated (1000 J/m^2^) firefly plasmid 250 ng/well using FuGene HD, according to the manufacturer’s protocol. After 48 h incubation, cell lysis, transfer to 96 well-plates and luminescence measurement using GloMax were performed according to the Dual-Luciferase Reporter Assay System protocol (Luciferase plasmids, Luciferase assay agents, transfection reagent and GloMax all from Promega, Madison, WI, USA). DNA repair capacities were determined by first calculating firefly to renilla luminescence ratios and then dividing irradiated to non-irradiated measurements. Finally, all calculated repair capacities were set in relation to those of A375 XPA wildtype (set as value 1).

### 4.7. 3-(4,5-dimethylthiazol-2-yl)-2,5-diphenyltetrazolium Bromide Tetrazolium (MTT) Assay

In total, 10,000 cells/well were seeded in 96 well-plates 24 h prior to UVC. Initial dosis-finding with increasing UVC irradiation was performed with A375 wildtype cells in between 0 and 500 J/m^2^. Non-UVC-treated cells and wells containing only DMEM served as control and blank, respectively. After 48 h incubation in 100 µL DMEM, 15 µL of MTT dye solution per well were added. After 4 h incubation 100 µL stop solution (MTT dye and stop solution from Promega, Madison, WI, USA) were added, and plates were incubated overnight. Absorptions were captured with Tecan Sunrise (Tecan Trading AG, Männedorf, Switzerland) and the differences between the readings of two wavelengths (550 nm and 650 nm) were indicative of measured cellular metabolic activity. All results were set in relation to the respective non-irradiated cells (set as value 1).

### 4.8. RNA Extraction

For every single experiment, 250,000 cells/well were seeded each in three wells of a 6-well plate as biological replicates. After 48 h incubation, total cellular RNA was extracted (RNeasy Mini Kit, Qiagen, Hilden, Germany) and the concentration was measured (NanoVue Plus, Biochrom, Thermo Fisher Scientific, Waltham, MA, USA).

### 4.9. Two-Step Quantitative Reverse Transcription PCR (RT-qPCR)

RNA was reverse transcribed at a concentration of 100 ng/µL (High Capacity cDNA Reverse Transcription Kit, Thermo Fisher Scientific, Waltham, MA), followed by 1:20 dilution. qPCR was performed in 384-well plates using QuantStudio™ 5 Real-Time PCR System (Thermo Fisher Scientific, Waltham, MA, USA) with 1 µL cDNA in a 10 µL reaction volume (PowerUp SYBR Green Master Mix, Thermo Fisher Scientific, Waltham, MA, USA) according to the manufacturer´s protocol. Primer sequences are deposited in Supplementary Table S1. Actin beta was used as housekeeping gene for normalization. ΔΔ*Ct* method was applied to analyze obtained data.

### 4.10. Statistical Analysis

Values are depicted as means ± SEM of data obtained from three independent experiments. Results were statistically analyzed using GraphPad Prism 8 (GraphPad Software, San Diego, CA, USA). Calculated relative DNA repair capacities, metabolic activity indicating cell survival and fold changes in mRNA expression were analyzed with one-way analysis of variance (ANOVA). *, **, *** indicate significance levels of *p* < 0.05, *p* < 0.01, *p* < 0.001, respectively.

## Data Availability

The original contributions presented in the study are included in the article/Supplementary Material. Further inquiries can be directed to the corresponding author.

## Supplementary Materials

The following supporting information can be downloaded at: https://www.mdpi.com/article/10.3390/ijms231911649/s1.
