# Supplementary material for: Homozygous CRISPR/Cas9 Knockout Generated a Novel Functionally Active Exon 1 Skipping XPA Variant in Melanoma Cells
Source: Int J Mol Sci. 2022 Oct 1;23(19):11649. doi: 10.3390/ijms231911649 (PMC9569948; doi:10.3390/ijms231911649)
Supplement: Supplementary file 1 [file ijms-23-11649-s001.zip › Figure S1.pdf]

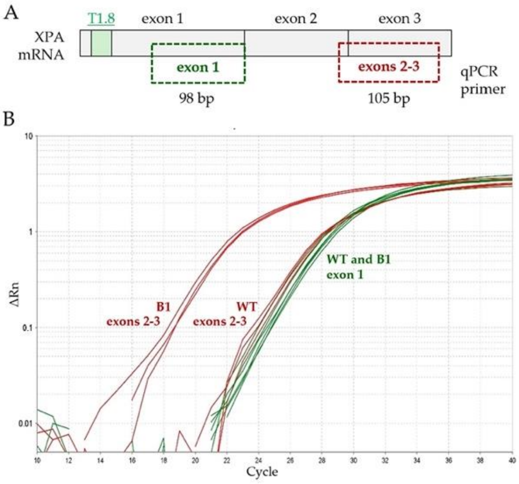

C

assay no.	target	WT			B1 (*c.19delG*)		
		sample 1	sample 2	sample 3	sample 1	sample 2	sample 3
1	*actin beta*	17.334	17.179	17.315	17.219	16.780	16.925
	XPA exon 1	26.465	26.612	26.031	25.999	26.520	27.132
	XPA exons 2-3	25.622	25.477	25.083	18.917	20.291	20.467
2	*actin beta*	17.253	16.947	16.938	17.772	16.889	17.708
	XPA exon 1	26.587	26.681	26.863	26.865	27.002	26.455
	XPA exons 2-3	25.028	25.585	25.637	20.975	20.586	20.292
3	*actin beta*	17.358	16.474	16.861	16.804	16.743	16.949
	XPA exon 1	27.291	26.751	26.470	26.338	26.870	25.808
	XPA exons 2-3	26.229	25.541	25.605	20.307	20.776	20.361

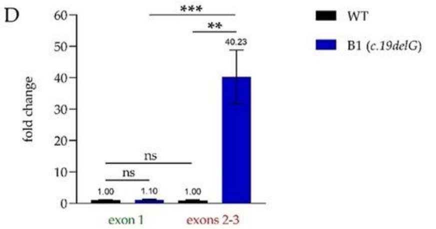

Supplementary Figure S1. qPCR of two different regions in *XPA*. (**A**) With two different primer pairs (dotted boxes), we amplified exon 1 downstream of the mutated region (highlighted in green, amplification product 98 bp) as well as the exon 2- 3 junction (in red, amplification product 105 bp). (**B**) Exemplary amplification plot. While detection of exon 1 (green curves) was similar in B1 and WT cells, we detected a large difference in the amplification of the exon 2 –3 spanning region (red curve). (C) Ct values are shown for actin beta as the housekeeping gene as well as for the two targeted regions of *XPA*. (**D**) Expression of exon 1 was similar in WT and B1 cells, while an about 40-fold increased expression of the region spanning exons 2-3 was manifest in B1. $2^{-\Delta\Delta Ct}$ values are depicted, normalization using actin beta. Significant differences are indicated by stars (* $p< .05$; ** $p< .01$; ***$p<.001$; one-way ANOVA). *Ns*, not significant; *ΔRn*, normalized reporter value.
