# Supplementary material for: Homozygous CRISPR/Cas9 Knockout Generated a Novel Functionally Active Exon 1 Skipping XPA Variant in Melanoma Cells
Source: Int J Mol Sci. 2022 Oct 1;23(19):11649. doi: 10.3390/ijms231911649 (PMC9569948; doi:10.3390/ijms231911649)
Supplement: Supplementary file 1 [file ijms-23-11649-s001.zip › Table S1.pdf]

Supplementary Table S1. Primers used for PCR and for qPCR of *XPA*. Amplified regions in gDNA/ mRNA, the amplicon lengths and primer sequences are given. *fwd*, forward; *gDNA*, genomic DNA; *mRNA*, messenger RNA; *rev*, reverse.

assay	amplified region	amplicon length		primer sequence 5′- 3′
PCR	gDNA XPA exon 1	592 bp	fwd	ccttctcccggatgacaaga
			rev	tggacaggacgctttgaca
	gDNA XPA exon 2	466 bp	fwd	gttagactagctgggaccttca
			rev	tgcatggctgtttctttgattg
	gDNA XPA exon 3	266 bp	fwd	caggcattgcatacatgctga
			rev	cttgttttgccctaaacctacaca
	gDNA XPA exon 4	371 bp	fwd	ctaagttgctgggctatttgc
			rev	gtgaagcatatgcaaaactaggg
	gDNA XPA exon 5	440 bp	fwd	gagttagatcattctttggtacctttg
			rev	taagatgatgacgtacatgcttttcag
	gDNA XPA exon 6	414 bp	fwd	ctgaaagcttgatggagttgg
			rev	ggtgaccttcactgaaacttgc
qPCR	mRNA XPA exon 1 *(after mutation)*	98 bp	fwd	ggcgagtatcgagcggaag
			rev	ctccagtagccgcagcc
	mRNA XPA exons 2-3 *(exon-exon junction)*	105 bp	fwd	tcatcaaccaggacctgttatg
			rev	caagttggcaaatcaaagtggt
